# Thermoelectric Properties of Cu_2_S Doped with P, As, Sb and Bi—Theoretical and Experimental Studies

**DOI:** 10.3390/ma17225440

**Published:** 2024-11-07

**Authors:** Paweł Nieroda, Juliusz Leszczyński, Krzysztof Kapera, Paweł Rutkowski, Krzysztof Ziewiec, Aleksandra Szymańska, Mirosław J. Kruszewski, Małgorzata Rudnik, Andrzej Koleżyński

**Affiliations:** 1 Department of Inorganic Chemistry, Faculty of Materials Science and Ceramics, AGH University of Krakow, al. A. Mickiewicza 30, 30-059 Krakow, Poland; jleszczy@agh.edu.pl (J.L.); aleksandra.szymanska5@gmail.com (A.S.); malrud@student.agh.edu.pl (M.R.); 2 Department of Silicate Chemistry and Macromolecular Compounds, Faculty of Materials Science and Ceramics, AGH University of Krakow, al. A. Mickiewicza 30, 30-059 Krakow, Poland; kaperak@agh.edu.pl (K.K.); andrzej.kolezynski@agh.edu.pl (A.K.); 3 Department of Ceramics and Refractories, Faculty of Materials Science and Ceramics, AGH University of Krakow, al. A. Mickiewicza 30, 30-059 Krakow, Poland; pawelr@agh.edu.pl; 4 Institute of Technology, University of the National Education Commission, Krakow, Podchorążych 2, 30-084 Krakow, Poland; krzysztof.ziewiec@up.krakow.pl; 5 Division of Materials Design, Faculty of Materials Science and Engineering, Warsaw University of Technology, Wołoska 141, 02-507 Warsaw, Poland; miroslaw.kruszewski@pw.edu.pl

**Keywords:** copper(I) sulfide, thermoelectric materials, spark plasma sintering (SPS), SPS melting, full-potential linearized augmented plane wave method, DFT calculations, electronic structure

## Abstract

The aim of this work was to investigate the possibility of doping copper sulfide Cu_2_S with selected fifth-group elements, potentially having a positive effect on the thermoelectric properties of the resulting materials. For the selected model structures, theoretical calculations and an analysis of the electronic structure and changes in the enthalpy of formation due to doping were performed using the WIEN2k package employing the Full-Potential Linearized Augmented Plane Wave (FP-LAPW) method within density functional theory (DFT) formalism. Polycrystalline materials with the nominal composition of Cu_32_S_15_X_1_ (*X* = P, As, Sb, Bi) were synthesized in quartz ampoules, then sintered using the spark plasma sintering (SPS) technique and “SPS melting” method. The chemical and phase compositions of the obtained sinters were studied by X-Ray diffraction (XRD) and scanning electron microscopy (SEM). Additionally, investigations of thermoelectric properties, i.e., electrical conductivity, Seebeck coefficient and thermal conductivity in the temperature range 300–920 K, were performed. The results of this study indicate that only phosphorus is successfully incorporated into the Cu₂S structure.

## 1. Introduction

**Figure 1:**
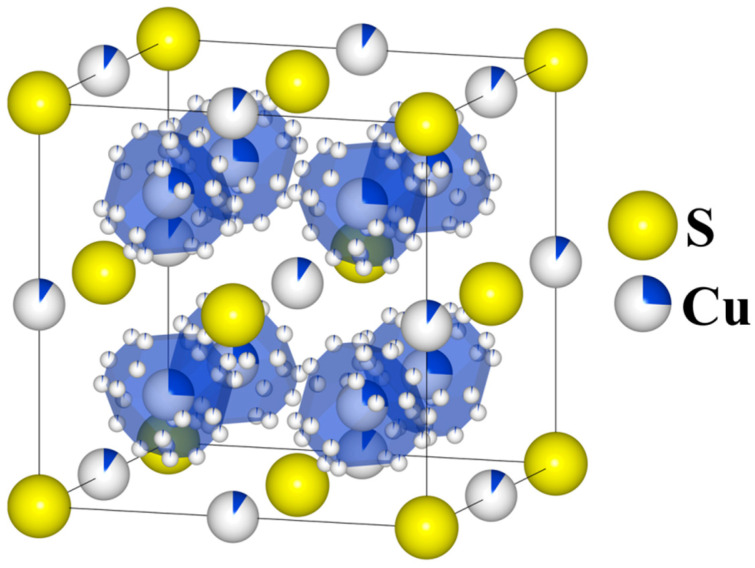
Model of real cubic Cu_2_S structure: Sulfur atoms occupy the 4*a* (0, 0, 0) Wyckoff position, while copper atoms partially occupy three symmetrically nonequivalent Wyckoff positions: the 8*c* (¼ ¼ ¼) position, with 24 possible sites surrounding each 8*c* position; the 192*l* (0.1067, 0.166, 0.2826) position (marked with polyhedral for clarity purposes) and the 4*b* (½, ½, ½) position in the middle of the edges.

Cu_2_S is a thermoelectric material and simultaneously superionic semiconductor with a very low thermal conductivity, resulting in high values of the *ZT* parameter [1,2,3], for which the concept of Phonon-Liquid Electron-Crystals (PLECs) described in [4] is applicable. The chalcocite Cu_2_S can assume one of three stable structures: the monoclinic (γ) phase (*T* < 103 °C), space group P21/c; the hexagonal (β) phase (103 °C < *T* < 435 °C), space group P63/mmc; and the cubic (α) phase (*T* > 435 °C), space group Fm3m [5]. Historically, determining the precise atomic positions for copper has been challenging because copper atoms are statistically distributed across multiple sites, with their distribution depending on temperature (Figure 1). At higher temperatures in the cubic phase, copper atoms are very mobile and can “jump” at short time intervals between equivalent crystal lattice sites (the partially occupied Wyckoff position 8*c*—which is typical of an calcium antifluoride structure—as well as 192*l* and 4*b* [6]), and thus its structure can be viewed as a rigid sulfur lattice “filled” with “liquid” copper.

Undoped Cu_2_S has an unsatisfying thermoelectric efficiency parameter *ZT* [7] (*ZT*_max_ = 0.68, *T* = 923 K [8], *ZT*_max_ = 0.32, *T* = 723 K [9], *ZT*_max_ = 0.58, *T* = 1000 K [10,11]); however, even a small deviation from the stoichiometry causes a significant increase in the *ZT* parameter, *e.g.*, *ZT*_max_ = 1.9, *T* = 973 K for Cu_1.97_S [12]. Another way of improving the thermoelectric properties of Cu_2_S that has been used successfully in recent years is the formation of solid solutions with copper(I) selenides and tellurides. The improvements in thermoelectric properties with the addition of Se can be attributed to two main factors: (a) the increase in carrier concentrations and their effective mass m*, which results in a higher power factor *PF* = α^2^σ, i.e., the product of electrical conductivity σ and the square of the Seebeck coefficient α; and (b) a decrease in both the lattice component of thermal conductivity and total thermal conductivity. This allowed a *ZT*_max_ = 0.74, *T* = 723 K to be achieved for Cu_2_S_1.9_Se_0.1_ [9]. An even more significant enhancement of the thermoelectric properties was achieved by alloying Cu_2_S and Cu_2_Te, which allowed the electrical conductivity to be increased by more than one order of magnitude with a simultaneous decrease in the Seebeck coefficient of about half, which, with a slight increase in thermal conductivity, made it possible to achieve *ZT*_max_ = 2.1, *T* = 1000 K for Cu_2_S_0.52_Te_0.48_ [10] and *ZT*_max_ = 2.1, *T* = 1000 K for Cu_2_S_0.94_Te_0.06_ [13]. However, such a substantial improvement in thermoelectric properties is suppressed by the formation of a mosaic nanostructure. The solid solution formation of Cu_2_S, Cu_2_Se and Cu_2_Te did not give the expected further improvement in thermoelectric properties compared to Cu_2_S_1-x_Te_x_, *ZT*_max_ = 1.5, *T* = 1000 K for Cu_2_S_1/3_Se_1/3_Te_1/3_ [14]; however, it allowed the thermoelectric properties to be better than in pure Cu_2_S and in Cu_2_S_1-x_Se_x_. There have also been attempts to improve Cu_2_S transport properties by doping at the Cu sites. In the case of doping with Li, an improvement in thermoelectric properties was achieved with *ZT*_max_ = 0.84, *T* = 900 K for Cu_1.99_Li_0.01_S [15], due to an increase in electrical conductivity with a small decrease in Seebeck coefficient and a slight increase in thermal conductivity. In another study [16], the effects of Mn, Zn, Ga and Ge dopants on Cu_1.95_X_0.03_S systems was investigated. For all the above dopants, an increase in electrical conductivity was observed (the highest for Manganese and the lowest for Germanium), with a simultaneous decrease in the Seebeck coefficient. The doping also caused an increase in thermal conductivity, but for all dopants it was still possible to obtain an increased *ZT* parameter. The Mn-doped sample showed the best properties but was comparable to undoped Cu_1.98_S (*ZT*_max_ = 0.91, *T* = 823 K). Despite all of the mentioned works, the number of studied dopants that could improve the thermoelectric properties of Cu_2_S is still relatively small. In the best case, the improvement in thermoelectric properties was comparable to copper-deficient Cu_2_S, so further studies devoted to transport property improvement via the respective doping methods are needed. Finding an effective dopant, besides improving the properties of pure Cu_2_S, could also facilitate the further fine-tuning of Cu_2_S-Cu_2_Te alloys. As follows from the literature review, doping in a S sublattice has not been studied before, but as shown in studies conducted for Cu_2_S-Cu_2_Se and Cu_2_S-Cu_2_Te systems, the substitution of S atoms with other atoms is possible.

The aim of this work was to investigate the possibility of doping copper sulfide in an anionic sublattice with selected fifth-group elements, potentially having a positive effect on the thermoelectric properties of the resulting materials.

## 2. Materials and Methods

### 2.1. Computational Details

**Figure 2:**
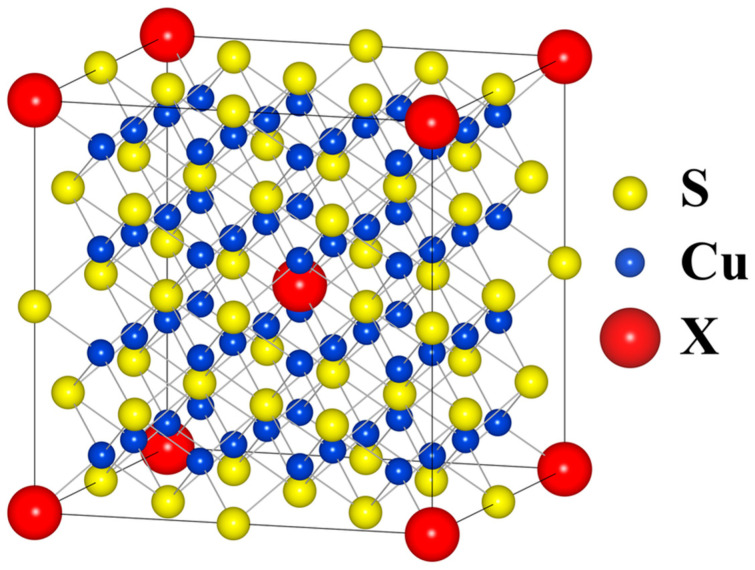
Model Fm3m 2 × 2 × 2 supercell structure used in calculations.

Ab initio calculations were performed using the WIEN2k package, which employs Full-Potential Linearized Augmented Plane Wave (FP-LAPW) approximation within density functional theory (DTF) formalism. All structures were fully relaxed and their internal parameters (unit cell parameters and atomic positions) were optimized with respect to the forces acting on atoms and the total energy of the system. PBESol generalized gradient approximation (GGA) was used as the exchange–correlation functional along with the convergence criteria of 10^−5^ Ry for energy, 5∙10^−4^ Ry/au for forces and 10^−3^ e for charge. To simulate Cu_32_S_15_X_1_ (where *X* = P, As, Sb, Bi), 2 × 2 × 2 supercells were created as shown in Figure 2. The positions of the dopant atoms were identical for all structures.

The total energy of relaxed structures allows us to calculate the enthalpy of formation, *H_F_*, which is defined as the energy difference between the total energy of a given structure and the sum of the total energies of the pure element structures in their thermodynamically most stable forms. For example, for Cu_32_S_15_As_1_, we have:

(1) $$H_{F}^{Cu_{32}S_{15}As_{1}}=E_{tot}^{Cu_{32}S_{15}As_{1}}-32\cdot E_{tot}^{Cu}-15\cdot E_{tot}^{S}-1\cdot E_{tot}^{As}$$

where both *H*_F_ and *E*_tot_ are in eV/atom. The structures of pure elements were also optimized and relaxed. Since the enthalpy of formation is a sign of the stability of a structure, one should compare the enthalpy of doping, which is the difference in the enthalpy of the formation of undoped and doped Cu_2_S.

WIEN2k also allows one to calculate the total and partial density of states (DOS) using the modified tetrahedron method [17]. However, because density functional theory is designed to describe ground state energies and one-electron eigenvalues are not suited to predicting one-electron excitations, the DFT calculations typically underestimate band gaps.

### 2.2. Experimental Details

Cu (99.9% Alfa Aesar, Kandel, Germany), S (99.999%, Alfa Aesar, Kandel, Germany), P (98.9%, Alfa Aesar, Kandel, Germany) and As powders (99%, Alfa Aesar, Kandel, Germany) and Bi and Sb ingots (99.5 and 99.99%, Alfa Aesar, Kandel, Germany) were used for the synthesis. A series of samples with the nominal composition Cu_32_S_15_X_1_ (*X* = P, As, Sb, Bi) and Cu_32_S_16-x_P_x_ (*x* = 0, 0.15, 0.25 and 1) were prepared by a direct two-step synthesis (I stage: *T*_1_ = 380 °C, *t*_1_ = 24 h, II stage: *T*_2_ = 900 °C, *t*_2_ = 18 h) from the above-mentioned elements in quartz ampoules, and sealed under a vacuum using a rocking furnace, similar to our previous works [8,18]. After each synthesis step, the obtained ingots were ground in an agate mortar. Next, the powders were densified in the SPS apparatus with an AC current, which does not cause the migration of copper ions, in contrast to the DC current, which causes the degradation of Cu_2_S, as shown in our previous work [18] (*T* = 500 °C, *p* = 65 MPa, *t* = 5 min, vacuum atmosphere *p* = 1∙10^−2^ mbar). Since the boiling point of phosphorus is 431 °C [19], the sintering of samples with phosphorus was carried out in a protective argon atmosphere (*p* = 1.4∙10^3^ mbar) to limit the possible release of unreacted phosphorus from the samples. Additionally, a modified SPS technique, which we named “SPS melting”, was employed. This method involves placing a special graphite container with powder in a graphite die, allowing for the melting and simultaneous densification of the synthesized samples (*T* = 1250 °C, *t* = 2 min). The density ρ of the samples was determined by Archimedes’ hydrostatic method and was higher than 99% of the theoretical density for the undoped samples. The obtained samples were characterized using the X-Ray powder diffraction method (XRD) (X-Ray Diffractometer Panalytical Empyrean, CuKα, *λ* = 1.5418 Å) and scanning electron microscopy (SEM) (FEI Nova NanoSEM 200 FEI Europe Company Scanning Electron Microscope equipped with an EDAX detector). The uniformity of the Seebeck coefficient of the samples was examined by a scanning thermoelectric microprobe (STM) at room temperature (tested area size 2 × 2 mm, 0.1 mm step) [20,21]. The thermoelectric properties were measured within the temperature range of 300 to 923 K using custom-made apparatus. Electrical conductivity *σ* was measured using alternating polarity DC current by the four-probe technique. During the measurements of the Seebeck coefficient *α*, a slight temperature gradient was applied (Δ*T* < 4 K). The thermal diffusivity *a* was measured and the specific heat *C*_p_ was determined by the laser flash method (LFA) (Laser Flash Apparatus LFA 427) in the temperature range 300–923 K. The thermal conductivity *λ* was calculated by *λ* = *a*∙*C*_p_∙ρ [22,23]. The thermoelectric figure of merit *ZT* parameter was assessed from the relationship *ZT* = *α*^2^*σλ*^−1^*T* [23,24,25,26].

## 3. Results and Discussion

### 3.1. Electronic Structure and Enthalpy of Doping

**Figure 3:**
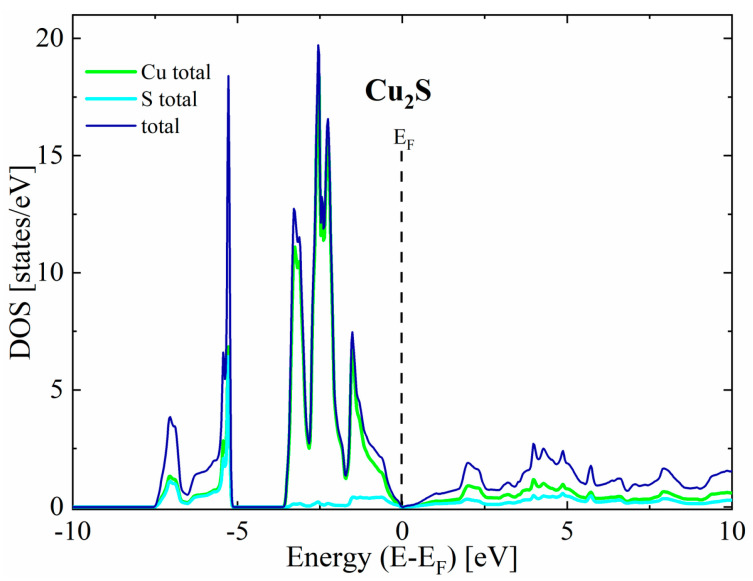
Partial density of states for pure Cu_2_S.

**Figure 4:**
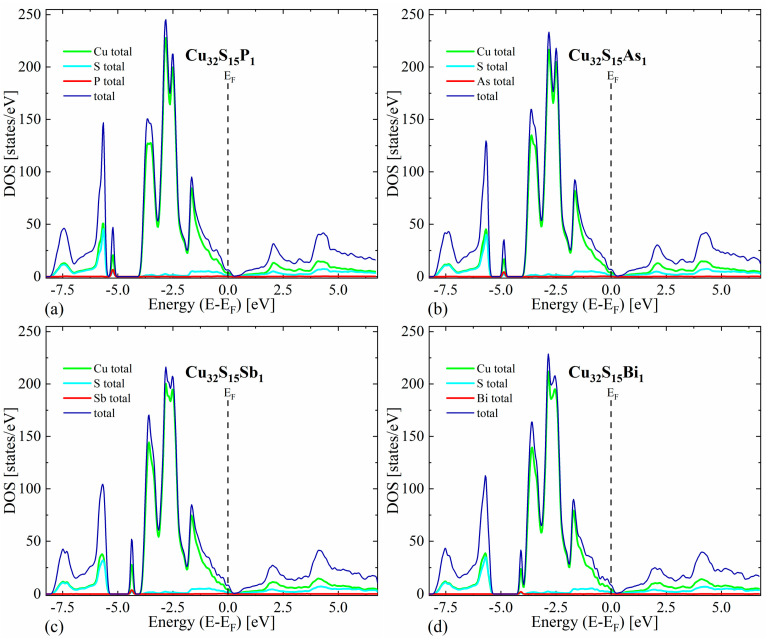
Density of states (total and projected onto particular atoms) calculated for Cu_2_S doped with (**a**) phosphor, (**b**) arsenic, (**c**) antimony and (**d**) bismuth occupying the sulfur position.

The first thing worth noting is that in all cases the valence bands close to the maximum have a predominant Cu character, while the conduction bands exhibit a more mixed character originating from both Cu and S states. For the undoped Cu_2_S, our calculations predict a metallic character without any band gap for the reasons explained in the theoretical details (Figure 3). In the case of doped structures, a shift in Fermi level toward lower energies can be seen, resulting in a non-zero density of states at the E_F_, indicating *p*-type semiconducting (Figure 4).

The negative values of enthalpy of doping means that the given structure is more favorable energetically than pure Cu_2_S (Table 1). It is, however, worth noting that in practice they might not necessarily form a real, doped material, since one cannot exclude the possibility that dopant atoms will energetically prefer to form other phases (sulfides, arsenides, bismuthinides and so on); nevertheless, predicting this is far beyond the scope of this work.

### 3.2. Structural and Microstructural Analysis

**Figure 5:**
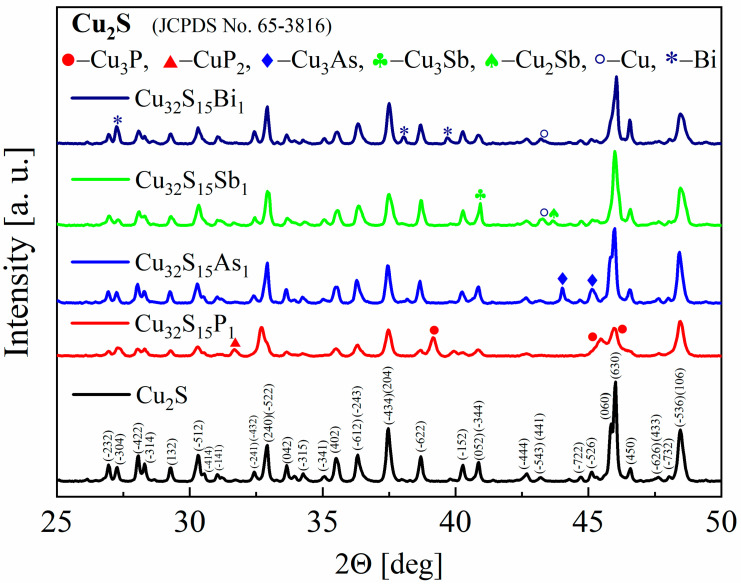
X-Ray diffraction patterns for Cu_2_S and Cu_32_S_15_X_1_ (*X* = P, As, Sb, Bi) samples after synthesis.

The XRD patterns of the synthesized powders as well as the sintered samples show that the materials obtained consist mostly of Cu_2_S with a monoclinic structure of chalcocite, a stable form of copper sulfide at room temperature. The appearance of secondary phases of copper, copper phosphides, antimonides and arsenides, and bismuth can also be observed for all samples (Figure 5), but they represent the vast minority of material. This means that not all of the dopant has been integrated into the structure and the dopant content has exceeded the solubility limit for each of the elements used. However, the accurate determination of the precipitate content is difficult due to a large number of Cu_2_S-derived reflections overlapping with the peak positions of the analyzed phases. The largest changes in the diffractogram appearance are observed for phosphorus doping. The intensities of the reflections undergo the greatest changes and the half-width of the reflections appear to be slightly enlarged. The main impurity observed is Cu_3_P, which is a typical phase in the Cu-P system [27]. For As doping, mainly Cu_3_As impurity peaks are observed in agreement with the phase diagram of the Cu-As system [28].

**Figure 6:**
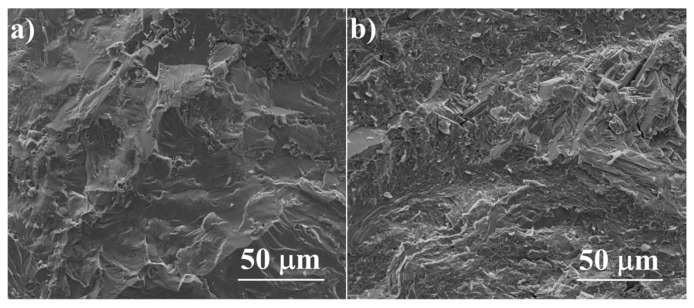
SEM photographs of surfaces of cross-section fractures for (**a**) Cu_2_S and (**b**) Cu_32_S_15_P_1_ samples after SPS.

The SEM observations combined with energy-dispersive X-Ray spectroscopy (EDS) analysis allow the determination, within the sensitivity of the EDS method, of the solubility of dopants in the material grains and the observation of precipitates in the obtained samples. All the samples have high density and there is no visible porosity (ρ > 99% of the theoretical density for undoped Cu_2_S), which is clearly visible in the sample fractures (Figure 6). All the obtained sinters are homogeneous and consist predominantly of a phase with a composition corresponding to Cu_2_S; however, minor precipitates of impurities are visible in each of the samples.

**Figure 7:**
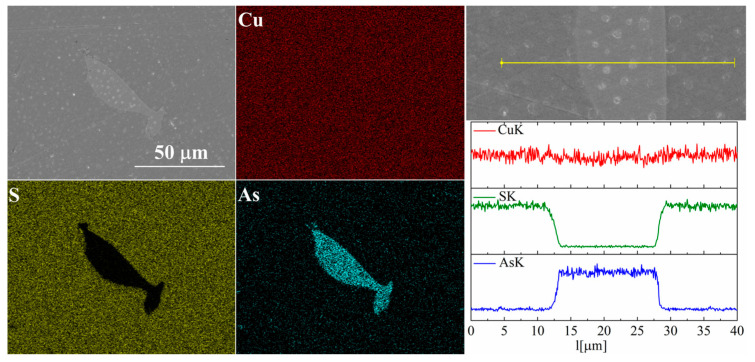
SEM photographs of selected surface with respective maps of element distribution and linear analyses of the chemical composition for Cu_32_S_15_As_1_ nominal composition after SPS.

**Figure 8:**
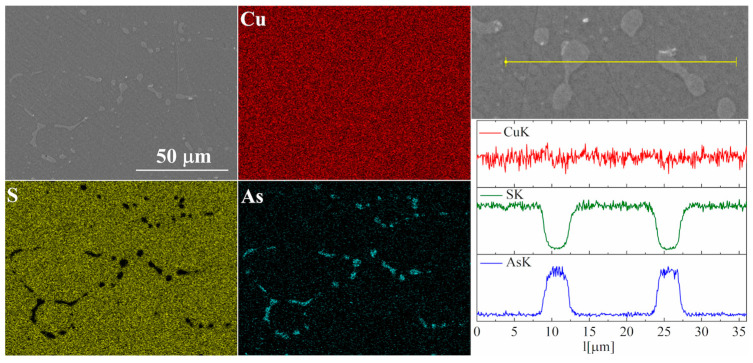
SEM photographs of selected surface with respective maps of element distribution and linear analyses of the chemical composition for Cu_32_S_15_As_1_ nominal composition after densification by the “SPS melting” method.

For the As-doped sample, a larger number of precipitates can be observed (Figure 7 and Figure 8). Most of these precipitates have a composition corresponding to Cu_3_As, which agrees with the XRD observations. Linear compositional analysis through the areas containing precipitates shows that there is hardly any sulfur in these precipitates, and the boundary between Cu_3_As and Cu_2_S is sharp and no enrichment of Cu_2_S with arsenic is observed near this boundary. The elemental distribution maps show that As is primarily accumulated in the precipitates. At the same time, point analysis did not show (within the sensitivity of the EDS method) any presence of As in the material grains.

**Figure 9:**
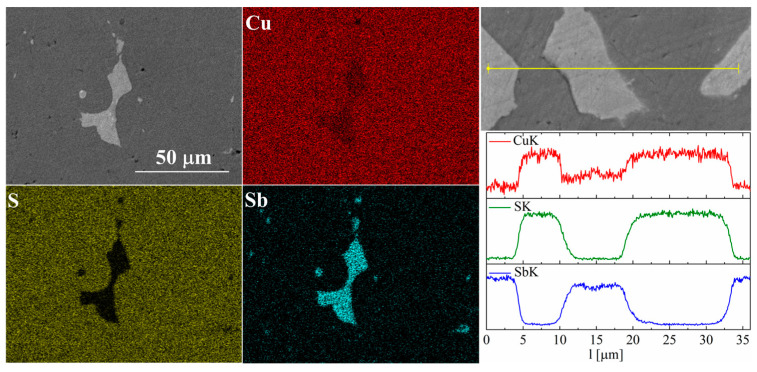
SEM photographs of selected surface with respective maps of element distribution and linear analyses of the chemical composition for the Cu_32_S_15_Sb_1_ nominal composition after SPS.

**Figure 10:**
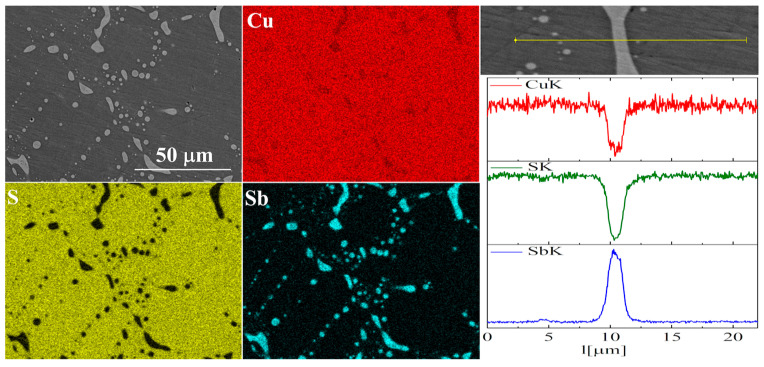
SEM photographs of selected surface with respective maps of element distribution and linear analyses of the chemical composition for Cu_32_S_15_Sb_1_ nominal composition after densification by “SPS melting” method.

In the case of Sb doping, SEM images show the presence of a large number of precipitates (Figure 9 and Figure 10). Composition analysis showed that most of these precipitates correspond to the Cu_3_Sb phase, and a minority are Cu_2_Sb. Chemical composition maps show that antimony is contained almost exclusively in the precipitates. A linear EDS analysis of the composition confirms these observations, showing that antimony is present exclusively in the precipitates, in which in turn sulfur is absent. A more detailed EDS point analysis of the composition showed that the Sb content in the Cu_2_S phase is close to zero and much smaller than the uncertainty of the measurement method.

**Figure 11:**
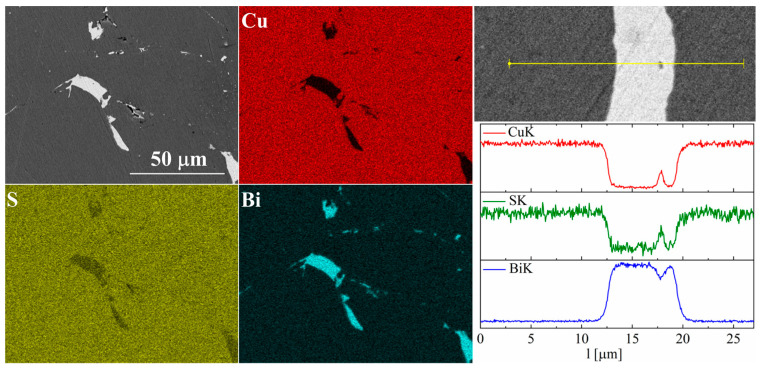
SEM photographs of selected surface with respective maps of element distribution and linear analyses of the chemical composition for Cu_32_S_15_Bi_1_ nominal composition after SPS.

**Figure 12:**
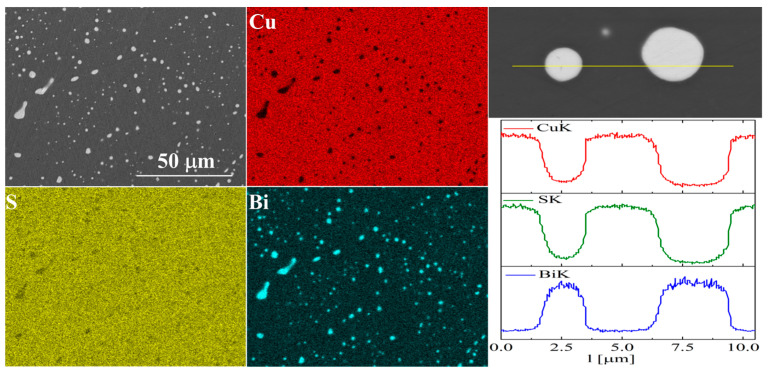
SEM photographs of selected surface with respective maps of element distribution and linear analyses of the chemical composition for Cu_32_S_15_Bi_1_ nominal composition after densification by “SPS melting” method.

For bismuth-doped Cu_2_S samples, the SEM observations also showed many impurity precipitates (Figure 11 and Figure 12). EDS compositional analysis shows that these are mostly bismuth precipitates. EDS linear analysis through the region containing bismuth precipitate showed that these precipitates were devoid of sulfur or copper. Careful EDS spot analysis in the Cu_2_S grains showed (within the sensitivity of this method) no Bi presence.

**Figure 13:**
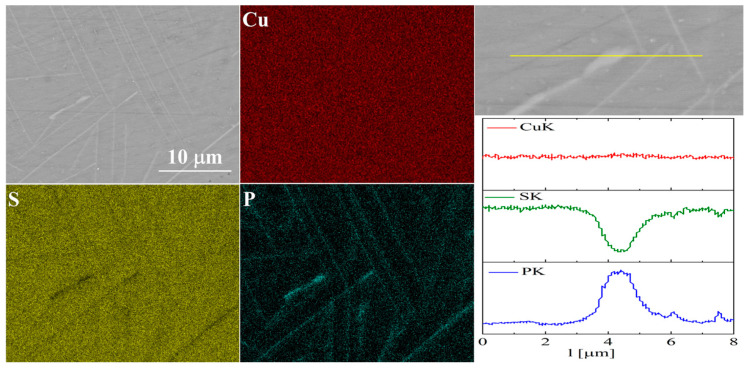
SEM photographs of selected surface with respective maps of element distribution and linear analyses of the chemical composition for Cu_32_S_15_P_1_ nominal composition after SPS.

**Figure 14:**
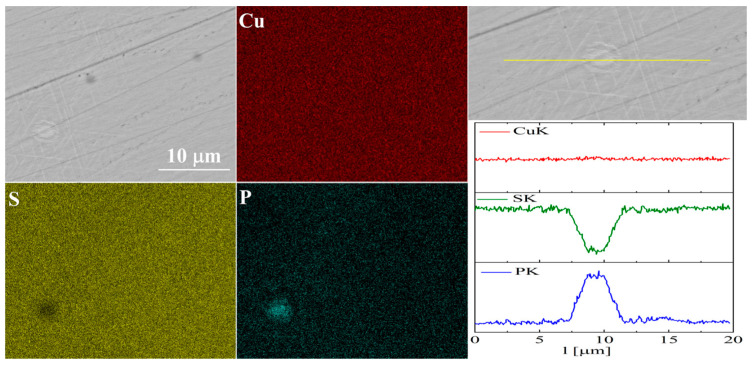
SEM photographs of selected surface with respective maps of element distribution and linear analyses of the chemical composition for Cu_32_S_15_P_1_ nominal composition after densification by “SPS melting” method.

The elemental distribution maps for Cu_32_S_15_P_1_ are shown in Figure 13 and Figure 14. In the case of Cu_2_S doped with phosphorus, precipitates rich in phosphorus and copper are visible, which is consistent with the XRD results.

Simultaneously, EDS point analysis in the material grains indicated a phosphorus content of 0.5–0.7 at.%, which is lower than the assumed nominal composition, i.e., about 2%. This is likely due to the formation of Cu_3_P, but this does not exclude the partial incorporation of P into the Cu_2_S structure. For this reason, phosphorus doping was considered the most promising, and additionally, samples with lower phosphorus content than in the initial one, i.e., *x* = 0.15 and 0.25 (for Cu_32_S_16-x_P_x_) were synthesized. Then, the materials were densified by the SPS method and the structural and thermoelectric properties examined.

**Figure 15:**
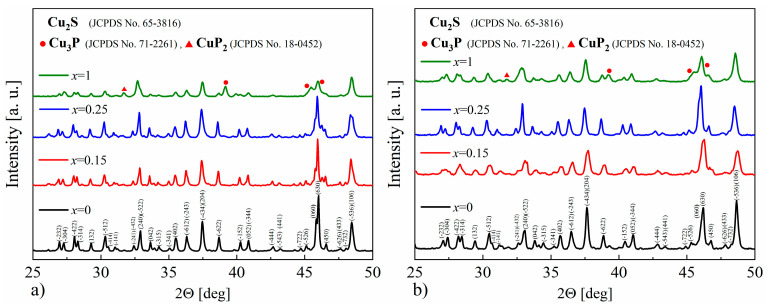
X-Ray diffraction patterns for Cu_32_S_16-x_P_x_ samples after (**a**) synthesis and (**b**) SPS.

The XRD results obtained for the Cu_32_S_16-x_P_x_ (*x* = 0–1) samples show that in each case, the dominant phase is the monoclinic Cu_2_S, and only in the sample with phosphorus content corresponding to *x* = 1 are the reflections originating from the Cu_3_P (Figure 15a,b) phase clearly visible. Based on the XRD results, the lattice parameters were determined using the Rietveld method, and in the case of P-doped samples, the cell volume was smaller compared to undoped Cu_2_S, which may indicate the successful phosphorus incorporation into the Cu_2_S structure. The densities of the samples for *x* = 0.15 and *x* = 0.25 are very similar, and equal to ρ = 5.67 ± 0.01 g∙cm^−3^ and ρ = 5.68 ± 0.01 g∙cm^−3^, respectively, and simultaneously lower compared to undoped Cu_2_S (ρ = 5.74 ± 0.01 g∙cm^−3^). On the other hand, for the *x* = 1 sample, the measured density is higher than for undoped copper sulfide (ρ = 5.77 ± 0.01 g∙cm^−3^), which is likely the result of the presence of the Cu_3_P phase (with a theoretical density of 7.37 g∙cm^−3^).

### 3.3. Thermoelectric Properties

**Figure 16:**
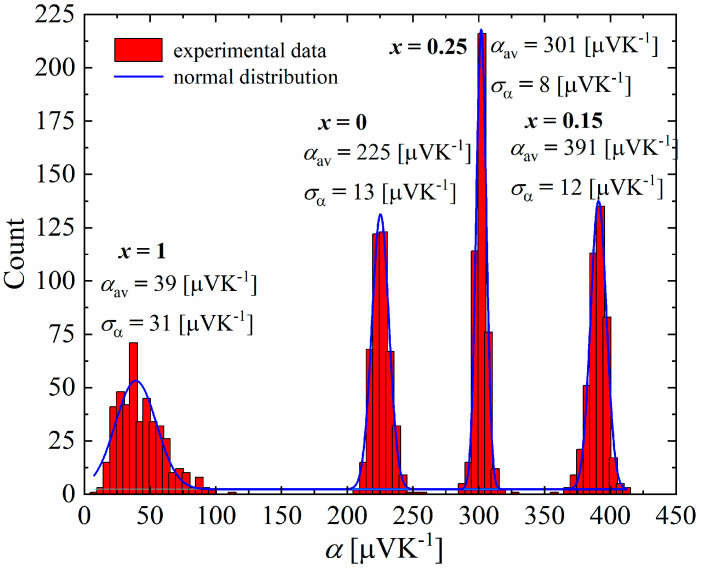
Seebeck coefficient distribution of Cu_32_S_16-x_P_x_ samples.

**Figure 17:**
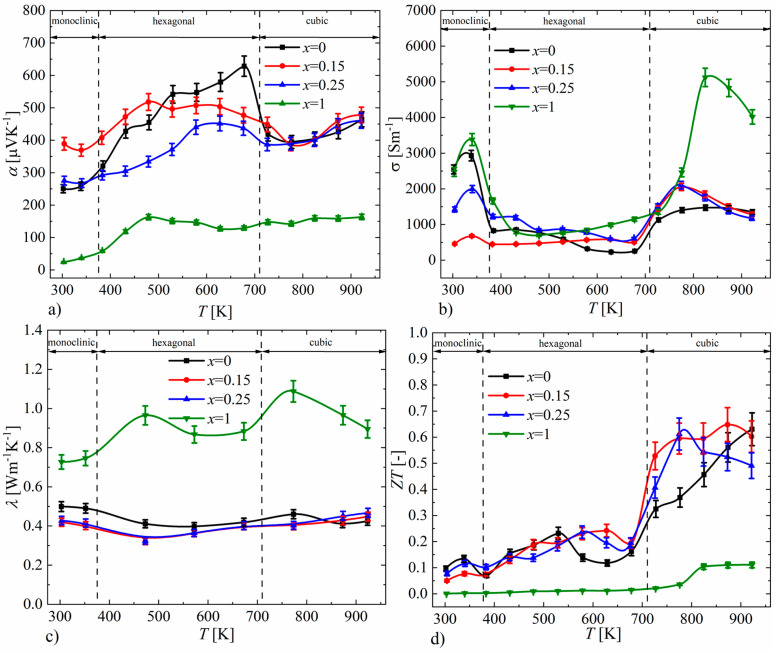
Temperature dependence of (**a**) electrical conductivity, (**b**) Seebeck coefficient, (**c**) thermal conductivity and (**d**) *ZT* parameter for Cu_32_S_16-x_P_x_ samples (absolute errors equal to 5% for α, σ, λ and 10% for *ZT*).

The studies carried out using a scanning thermoelectric microprobe (STM) showed that all samples have a unimodal distribution of the Seebeck coefficient (Figure 16), with a clearly visible difference between the mean values and standard deviations of the samples for x = 0–0.25 and *x* = 1, which appears to be related to the presence of an additional Cu_3_P phase, which has a very low Seebeck coefficient at room temperature (α = 11.2 μV∙K^−1^ for the Cu_3_P polycrystalline thin films [29], α = 9,85 μV∙K^−1^ theoretically calculated [30]). Samples in the range of x = 0–0.25 exhibit a narrow distribution of the Seebeck coefficient, with standard deviations equal to α_σ_ = 13 μV∙K^−1^ for *x* = 0, α_σ_ = 12 μV∙K^−1^ for *x* = 0.15 and α_σ_ = 8 μV∙K^−1^ for *x* = 0.25. This indicates the high homogeneity of these materials. The mean values of the Seebeck coefficient obtained in STM measurements were qualitatively consistent with the measurements of this parameter for bulk samples at room temperature (Figure 17).

The results of the measurements of the Seebeck coefficient as a function of temperature for P-doped Cu_2_S samples are presented in Figure 17a. The positive values of the Seebeck coefficient for all samples indicate that the obtained materials are *p*-type semiconductors. In the case of samples for *x* = 0.15 and *x* = 0.25, the character of the Seebeck coefficient course is similar to that of undoped Cu_2_S, while in the cubic phase region (*T* > 710 K), which is particularly interesting from the perspective of thermoelectric properties, samples in the range of *x* = 0–0.25 exhibit very similar values. However, for the sample with *x* = 1, the Seebeck coefficient values are significantly lower compared to the undoped sample over the entire tested temperature range. The electrical conductivity (Figure 17b) for samples with *x* = 0.15 and *x* = 0.25, like the Seebeck coefficient, has values similar to those of undoped Cu_2_S, while the *x* = 1 sample shows significantly higher values, especially in the high-temperature cubic phase region. The course of thermal conductivity values (Figure 17c) for samples doped with phosphorus is consistent with the results of the Seebeck coefficient and electrical conductivity; the samples with *x* = 0.15 and *x* = 0.25 show similar values to those of the undoped sample, while the *x* = 1 sample has significantly higher thermal conductivity values across the entire tested temperature range. The clear differences in the thermoelectric properties of the sample with *x* = 1 compared to the samples with a lower nominal amount of phosphorus dopant seem to be an effect of the presence of the Cu_3_P phase, which is the result of significantly exceeding the solubility limit of P dopant in Cu_2_S. The thermoelectric efficiency parameter *ZT* determined from the transport property measurements is highest for the undoped Cu_2_S sample (*ZT*_max_ = 0.63, *T* = 923 K), which is slightly higher than that of the *x* = 0.15 sample (*ZT*_max_ = 0.6, *T* = 923 K). It is also noteworthy that the average *ZT* value for the high-temperature cubic phase is greater for the *x* = 0.15 sample compared to the *x* = 0 sample (Figure 17d).

## 4. Conclusions

High-temperature synthesis and material densification employing the SPS of copper(I) sulfide doped with fifth-group elements, with the general formula Cu_32_S_15_X_1_ (*X* = P, As, Sb, Bi) and Cu_32_S_16-x_P_x_ (*x* = 0, 0.15, 0.25 and 1), were carried out in this study. All obtained materials had the dominant monoclinic phase typical for copper(I) sulfide. The chemical composition of the obtained samples was examined by point and line analysis using SEM (scanning electron microscopy). Within the sensitivity limits of the method, the solubility of Sb, Bi and As dopants in the Cu_2_S structure was not confirmed. The low solubility of P was indicated; however, despite its observed slight presence in the material grains, it was also found in the precipitates, forming compounds with copper. Only in the case of phosphorus was a small amount in the material grains detected, which indicates its solubility in the Cu_2_S structure. The theoretical analysis of the electronic structure showed that Cu_2_S doped with phosphorus should be a *p*-type semiconductor, which was confirmed by the experimental results of the Seebeck coefficient measurements. The phosphorus dopants were successfully introduced into the Cu_2_S structure for *x* = 0.15 and *x* = 0.25, and have similar thermoelectric properties compared to the undoped material, with the average values of the *ZT* parameter for the sample with *x* = 0.15 being higher than for the undoped Cu_2_S for the high-temperature cubic phase.

## Figures and Tables

**Table 1:** Enthalpy of doping for Cu_32_S_15_X_1_ (*X* = P, As, Sb, Bi).

Model Structure	Enthalpy of Doping ∆*H* [eV/atom]
Cu_32_S_15_P_1_	−0.00682
Cu_32_S_15_As_1_	0.00722
Cu_32_S_15_Sb_1_	0.02018
Cu_32_S_15_Bi_1_	−0.21230

## Data Availability

The original contributions presented in the study are included in the article and Supplementary Material, further inquiries can be directed to the corresponding author.

## Supplementary Materials

The following supporting information can be downloaded at: https://www.mdpi.com/article/10.3390/ma17225440/s1. Figure S1. Temperature dependence of lattice thermal conductivity for Cu_32_S_16-x_P_x_ samples (*L* = 2.44 ∙ 10^−8^ WΩK^−2^). Figure S2. Temperature dependence of specific heat for Cu_32_S_16-x_P_x_ samples and for undoped Cu_2_S for other authors [11,12,31].

## References

[1] Liu W.-D., Yang L., Chen Z.-G., Zou J. (2020). Promising and eco-friendly Cu_2_X-based thermoelectric materials: Progress and applications. Adv. Mater..

[2] Zhao K., Qiu P., Shi X., Chen L. (2020). Recent Advances in Liquid-Like Thermoelectric Materials. Adv. Funct. Mater..

[3] Qiu P., Shi X., Chen L. (2016). Cu-based thermoelectric materials. Energy Storage Mater..

[4] Liu H., Shi X., Xu F., Zhang L., Zhang W., Chen L., Li Q., Uher C., Day T., Snyder J. (2012). Copper ion liquid-like thermoelectrics. Nat. Mater..

[5] Chakrabarti D.J., Laughlin D.E. (1983). The Cu-S (Copper-Sulfur) system. Bull. Alloy Phase Diagr..

[6] Will G., Hinze E., Rahman A., Abdelrahman M. (2002). Crystal structure analysis and refinement of digenite, Cu_1.8_S, in the temperature range 20 to 500 °C under controlled sulfur partial pressure. Eur. J. Miner..

[7] Uher C. (2016). Materials Aspect of Thermoelectricity.

[8] Nieroda P., Leszczyński J., Mikuła A., Mars K., Kruszewski M.J., Koleżyński A. (2020). Thermoelectric properties of Cu_2_S obtained by high temperature synthesis and sintered by IHP method. Ceram. Int..

[9] Yao Y., Zhang B.-P., Pei J., Liu Y.-C., Li J.-F. (2017). Thermoelectric performance enhancement of Cu_2_S by Se doping leading to a simultaneous power factor increase and thermal conductivity reduction. J. Mater. Chem. C.

[10] He Y., Lu P., Shi X., Xu F., Zhang T., Snyder J., Uher C., Chen L. (2015). Ultrahigh thermoelectric performance in mosaic crystals. Adv. Mater..

[11] He Y., Day T., Zhang T., Liu H., Shi X., Chen L., Snyder J.G. (2014). High thermoelectric performance in non-toxic earth abundant copper sulfide. Adv. Mater..

[12] Zhao L., Xiaolin W., Fei F.Y., Wang J., Cheng Z., Dou S., Wang J., Snyder G.J. (2015). High thermoelectric and mechanical performance in highly dense Cu_2−x_S bulks prepared by a meltsolidification technique. J. Mater. Chem. A.

[13] Yao Y., Zhang B.-P., Pei J., Sun Q., Nie G., Zhang W.-Z., Zhuo Z.-T., Zhou W. (2018). High Thermoelectric Figure of Merit Achieved in Cu_2_S_1−x_Te_x_ Alloys Synthesized by Mechanical Alloying and Spark Plasma Sintering. ACS Appl. Mater. Interfaces.

[14] Zhao K., Zhu C., Qiu P., Blichfeld A.B., Eikeland E., Ren D., Iversen B.B., Xu F., Shi X., Chen L. (2017). High thermoelectric performance and low thermal conductivity in Cu_2−y_S_1/3_Se_1/3_Te_1/3_ liquid-like materials with nanoscale mosaic structures. Nano Energy.

[15] Guan M.-J., Qiu P.-F., Song Q.-F., Yang J., Ren D.-D., Shi X., Chen L.-D. (2018). Improved electrical transport properties and optimized thermoelectric figure of merit in lithium-doped copper sulfides. Rare Met..

[16] Liang X., Jin D., Dai F. (2019). Phase Transition Engineering of Cu_2_S to Widen the Temperature Window of Improved Thermoelectric Performance. Adv. Electron. Mater..

[17] Blöchl P.E., Jepsen O., Andersen O.K. (1994). Improved tetrahedron method for Brillouin-zone integrations. Phys. Rev. B.

[18] Nieroda P., Kruszewski M.J., Leszczyński J., Mars K., Koleżyński A. (2023). Influence of DC and AC current in the SPS sintering process on homogeneity of thermoelectric properties of Cu_2_S and Cu_2_Se. Ceram. Int..

[19] Honig R.E. (1957). Vapor Pressure Data For The More Common Elements. RCA Rev..

[20] Nieroda P., Kutorasinski K., Tobola J., Wojciechowski K.T. (2014). Search for resonant-like impurity in Ag-doped CoSb_3_ skutterudite: Theoretical and experimental study. J. Electron. Mater..

[21] Platzek D., Karpinski G., Stiewe C., Ziolkowski P., Drasar C., Müller E. Potential Seebeck-microprobe (PSM): Measuring the spatial resolution of the Seebeck coefficient and the electric potential. Proceedings of the ICT 2005 24th International Conference on Thermoelectrics.

[22] Min S., Blumm J., Lindemann A. (2007). A new laser flash system for measurement of the thermophysical properties. Thermochim. Acta.

[23] Shinzato K., Baba T. (2001). A Laser Flash Apparatus for Thermal Diffusivity and Specific Heat Capacity Measurements. J. Therm. Anal. Calorim..

[24] Snyder J., Toberer E.S. (2008). Complex thermoelectric materials. Nat. Mater..

[25] Giri K., Wang Y.-L., Chen T.-H., Chen C.-H. (2022). Challenges and strategies to optimize the figure of merit: Keeping eyes on thermoelectric metamaterials. Mater. Sci. Semicond. Process..

[26] Kumar R., Singh R. (2021). Thermoelectricity and Advanced Thermoelectric Materials.

[27] Predel B., Madelung O. (1994). Cu-P (Copper-Phosphorus). Landolt-Börnstein—Group IV Physical Chemistry.

[28] Subramanian P.R., Laughlin D.E. (1988). The As-Cu (Arsenic-Copper) System. Bull. Alloy Phase Diagr..

[29] Crovetto A., Unold T., Zakutayev A. (2023). Is Cu_3−x_P a Semiconductor, a Metal, or a Semimetal?. Chem. Mater..

[30] Choudhary K., Garrity K.F., Tavazza F. (2020). Data-driven discovery of 3D and 2D thermoelectric materials. J. Phys. Condens. Matter.

[31] Liang X. (2017). Mobile copper ions as heat carriers in polymorphous copper sulfide superionic conductors. Appl. Phys. Lett..

