# Supplementary material for: Thermoelectric Properties of Cu2S Doped with P, As, Sb and Bi—Theoretical and Experimental Studies
Source: Materials (Basel). 2024 Nov 7;17(22):5440. doi: 10.3390/ma17225440 (PMC11595463; doi:10.3390/ma17225440)
Supplement: Supplementary file 1 [file materials-17-05440-s001.zip › materials-3260736-supplementary.pdf]

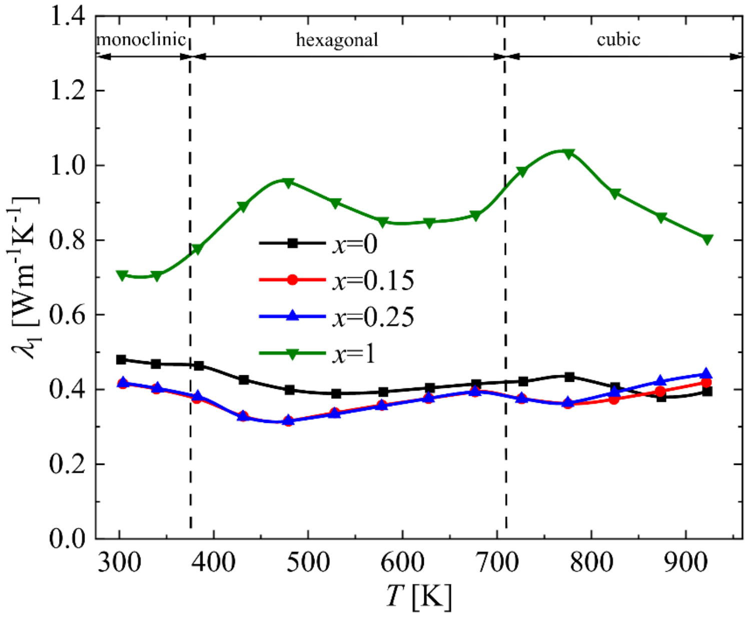

Figure S1. Temperature dependence of lattice thermal conductivity for $Cu_{32}S_{16-x}P_x$ samples (L = 2.44 · 10^{-8} WΩK^{-2}).

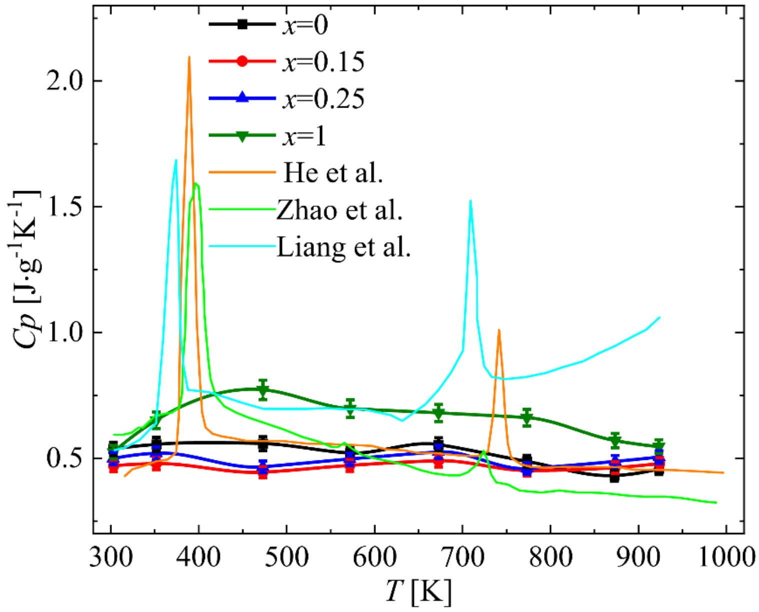

Figure S2. Temperature dependence of specific heat for $Cu_{32}S_{16-x}P_x$ samples and for undoped Cu_2S for other authors [1–3].

References

1. He, Y.; Day, T.; Zhang, T.; Liu, H.; Shi, X.; Chen, L.; Snyder, J.G. High thermoelectric performance in non-toxic earth abundant copper sulfide. *Adv. Mater.* **2014**, *26*, 3974–3978.
2. Zhao, L.; Xiaolin, W.; Fei, F.Y.; Wang, J.; Cheng, Z.; Dou, S.; Wang, J.; Snyder, G.J. High thermoelectric and mechanical performance in highly dense $Cu_{2-x}S$ bulks prepared by a meltsolidification technique. *J. Mater. Chem. A* **2015**, *3*, 9432–9437.
3. Liang, X. Mobile copper ions as heat carriers in polymorphous copper sulfide superionic conductors. *Appl. Phys. Lett.* **2017**, 111, 133902.
